# Influence of Multi-Cue Interaction on Human Depth Perception in Three-Dimensional Space

**DOI:** 10.3390/s26020413

**Published:** 2026-01-08

**Authors:** Qiang Liu, Shuai Li, Qiang Yang, Caihong Dai, Shufang He, Hiroaki Shigemasu

**Affiliations:** 1 Academy of Artificial Intelligence, Beijing Institute of Petrochemical Technology, Beijing 102627, China; 2 Division of Optical Metrology, National Institute of Metrology, Beijing 100029, China; 3 School of Informatics, Kochi University of Technology, Kami City 782-8502, Kochi, Japan

**Keywords:** 3D display, depth perception, motion parallax, color, position effect, binocular disparity

## Abstract

Background: With the widespread application of three-dimensional (3D) display technology, enhancing the realism of users’ experience in virtual 3D space has become important. A deep understanding of the mechanisms of human depth perception is therefore crucial. Objective: This study aims to investigate the influence of motion parallax, color, and object position cues on depth perception in 3D space. Method: Random-dot stereograms based on binocular disparity cues were constructed; three experiments were designed, varying the stimulus movement speed, color, and position; two-alternative forced-choice (2AFC) psychophysical paradigms were employed to collect participants’ responses regarding depth perception; and statistical analyses were conducted to examine the influences of these three cues on depth perception specified by binocular disparity. Results: A relatively small amount of motion parallax indicated a certain inhibitory effect on depth perception, whereas a larger amount might enhance the perceived depth. Introducing red, green, or blue color to the moving stimuli might also have a certain promoting effect. Furthermore, a significant difference in perceived depth was observed when the positions of the Test Stimulus and the Standard Stimulus differed within a trial, which might involve areas of higher-level brain function (such as visual attention). In conclusion, when multiple visual cues are present concurrently, they exhibit complex interactions that affect human depth perception.

## 1. Introduction

With the rapid development of three-dimensional (3D) display technology, immersive technologies such as Virtual Reality (VR), Augmented Reality (AR), and 3D films have become key development directions in many fields, including entertainment, healthcare, education, military simulation, and industrial design. The popularization of this technology has further promoted users’ demand for realistic navigation and interaction in virtual environments. The core of achieving high-quality immersive experiences lies in the accurate perception of depth in 3D space, which remains a key challenge and research focus in the development of 3D systems. If depth information is not accurately presented, it will cause problems such as visual fatigue and spatial disorientation, and create security risks in critical applications. Therefore, a thorough understanding of the mechanism of human depth perception is of vital significance for optimizing 3D display technology and enhancing user experience.

The human visual system can perceive 3D spatial information by fusing various visual cues (such as binocular disparity, motion parallax, color, and spatial position) through two-dimensional retinal images obtained by the left and right eyes [1,2,3]. Among them, binocular disparity stems from a horizontal distance of approximately 6 to 7 cm between the two eyes, which causes subtle differences in the imaging positions of external objects on the left and right retinas. The brain calculates the relative and absolute distances of objects in 3D space by fusing these two images and analyzing their positional differences. This visual mechanism plays a key role in near-field depth perception. Most types of 3D display devices are also designed based on this principle.

Motion parallax is an important monocular depth cue that occurs when the observer moves their head or when objects in the scene move. This phenomenon is manifested as objects at different distances moving at different speeds on the retina: closer objects appear to move faster, while those that are further away move more slowly. This cue constitutes a dynamic scene and plays an important role in distant scenes where the binocular disparity effect is weak [4]. Research on motion parallax as a depth cue can be traced back to the 19th century. With the invention of the stereoscope, Wheatstone pointed out that even without binocular disparity, head movement could produce equivalent depth perception [5]. In 1925, Helmholtz defined such cues as motion parallax and pointed out that it could induce a depth perception effect similar to that of binocular disparity [6]. Although subsequent studies have questioned its effectiveness, Rogers and Graham constructed an experimental paradigm of observer or object motion through random-dot stereograms, proving that motion parallax can serve as an independent and effective depth cue [7]. De la Malla et al. found that even a few millimeters of slight head movement could significantly affect depth perception by designing different types of motion parallax cues [8]. Fulvio et al. further confirmed that head shaking can affect depth perception, a phenomenon that was not detected in most experiments with fixed heads [9]. Furthermore, Dokka et al. discovered, based on motion parallax cues, that both the observer’s movement speed and the retinal image’s movement speed have an impact on depth perception [10].

Color, as another important visual cue, has also drawn much attention due to its role in depth perception. However, the relevant mechanisms are rather complex. A well-known phenomenon is “chromo-stereoscopy”; that is, when observed from the same distance, objects of different colors appear to have different depths. This phenomenon refers to the fact that even if objects of different colors are at the same physical distance, their visually perceived depth will vary [11]. Studies have shown that warm tones with longer wavelengths in the spectrum (such as red and yellow) are often perceived as being closer, while cool tones with shorter wavelengths (such as blue and green) appear further away [12]. However, color is not an independent depth cue but interacts with factors such as brightness, contrast, background environment, and the tone itself in a complex way [13]. For instance, objects with high contrast usually seem closer. In addition, the contribution of different tones to depth is also not equal. Chen et al.’s research systematically compared the disparity fusion range of different tonal stimuli. The results show that the yellow range is the largest, followed by red and blue, and the green range is the smallest [14]. This indicates that under the condition of the same disparity, the human eye’s perception of the depth of different colors varies.

The geometric position of objects in a scene is another key factor influencing human depth perception, and its effect does not rely on retinal or physiological factors. Research shows that in scenes containing visible ground planes or architectural structures, observers usually consider objects at lower positions in the frame that have optical contact with the ground to be closer, while those closer to the horizon or significantly detached from the ground are regarded as further away [15]. When the height of the vertical image and the distance to the horizon are separated in the experiment, the latter usually predicts the depth sequence more reliably, indicating that observers tend to infer the distance based on the projected position of the object on the ground plane and its relationship with the horizon [16]. This principle is consistent with J.J. Gibson’s ground theory, which holds that a continuous ground is the main reference framework for organizing spatial layout [17,18]. Therefore, when an object is depicted as floating above the ground or above the horizon, the stability of depth judgment decreases and the error rate increases accordingly. Even with the introduction of motion parallax and shading cues, such location-based depth perception effects still exist [18].

Regarding how the above-mentioned cues affect depth perception, although there are some research results at present, there are still several key issues that need to be further explored. Firstly, most existing studies have focused on a single depth cue and have not systematically revealed the interaction mechanisms among multiple cues, such as motion parallax and binocular disparity. Secondly, although color stereo vision can cause differences in depth perception, there is still a lack of systematic research on how color information affects depth perception under controlled motion conditions. This issue is particularly important in the context of modern display technologies with a wide color gamut and high contrast. Furthermore, the internal mechanism by which the spatial position of objects in the field of vision affects depth perception remains unclear at present.

To further clarify the above issues, this paper will systematically explore the influence of cues such as motion parallax, color, and the position of stimuli on depth perception by constructing three sets of psychophysical experimental paradigms. This study is expected to make contributions in both theory and practice. At the theoretical level, this paper will obtain experimental data on the influence of motion speed, color, and the position of the stimulus on depth perception, and analyze the influence of each cue in the depth perception of the stimulus constructed based on binocular disparity cues. At the application level, this article aims to provide data support for enhancing the depth experience in VR, AR, and 3D displays.

## 2. Materials and Methods

### 2.1. Participants

The experiments were conducted at the National Institute of Metrology, China. Experiment 1 was completed by 11 participants aged 24 to 37 years (5 males, mean age 30.1 years); Experiment 2 involved 10 participants aged 24 to 37 years (5 males, mean age 29.6 years); and Experiment 3 was carried out by 8 participants aged 24 to 35 years (3 males, mean age 30.3 years). All participants possessed normal or corrected-to-normal visual acuity and were naïve to the specific purpose of the experiment. All recruited participants successfully passed a stereoacuity test (stereoacuity better than 1 arcmin) and a color vision test. The details of the screening procedure are described in Section 3.1. All experiments and procedures were approved by the Research Ethics Committee of the National Institute of Metrology and conformed to the tenets of the Declaration of Helsinki. Written informed consent was obtained from all participants prior to experiments.

### 2.2. Apparatus

The experiments were conducted in a dark room to eliminate ambient light interference. Visual stimuli were presented on a 27-inch LCD monitor (Legion Y27gq-20; Lenovo, Beijing, China) with a spatial resolution of 1920 × 1440 pixels and a refresh rate of 120 Hz. Prior to data collection, we performed gamma correction by measuring the screen luminance with a CS-2000A colorimeter (Konica Minolta, Tokyo, Japan) and applying a look-up table (LUT) for linearization [19].

Stimuli were generated and controlled using MATLAB (Version R2022b, MathWorks, Natick, MA, USA) with the Psychophysics Toolbox extensions (Version 3) [20,21]. Participants observed the stimuli through liquid crystal shutter glasses (3D Vision 2; NVIDIA, Santa Clara, CA, USA). The liquid crystal shutter glasses were synchronized with the display refresh rate via a USB-connected infrared emitter (3D Vision USB IR emitter; NVIDIA, Santa Clara, CA, USA). Given the monitor’s 120 Hz refresh rate, the effective frame rate was 60 Hz per eye, providing a stable stereoscopic view with no reported flicker. A chin rest was used to stabilize the participant’s head position and strictly maintain the viewing distance. Participants judged the observed depth information, and made selections via specific keys on a keyboard.

### 2.3. Stimuli

**Figure 1:**
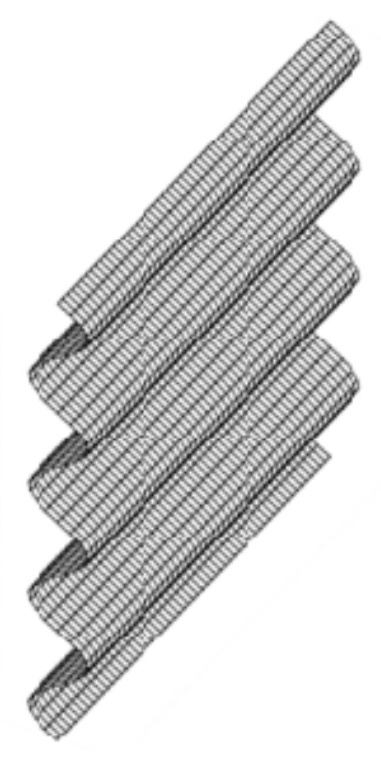
Experimental stimulus.

The experimental stimuli were random-dot stereograms. In Experiments 1 and 3, white random dots (29.7 cd/m^2^) were presented on a gray background (9.9 cd/m^2^). In Experiment 2, random dots (29.7 cd/m^2^) in four colors (white, red, green, and blue) were presented on the same gray background. Random-dot stereograms based on binocular disparity were presented by offsetting the images for the left and right eyes to generate a 3D sinusoidal corrugation surface with a specific depth (Figure 1). For the motion parallax 3D stimuli, the disparity was calculated to move the stimuli within a specific screen area at a specific speed. The use of random dots as stimuli effectively eliminated the influence of other cues on depth perception.

A Nonius fixation, consisting of an upright T and an inverted T, was displayed randomly on either the left or right side of the screen. Participants were instructed to fuse the two shapes by aligning the vertical segments and superimposing the horizontal bars to form a perfect cross. This alignment task ensured proper eye vergence throughout the experiment. Both the horizontal and vertical components of the stimulus subtended 1.17 arcdeg [19].

The dimensions of the corrugation pattern were 13.5 cm (vertical) × 13.5 cm (horizontal). Under binocular disparity conditions, the peak amplitude of the 3D corrugation was 6.3 arcmin. The viewing distance was set to 57 cm, and the luminance was 30.0 cd/m^2^.

## 3. Experimental Procedure

### 3.1. Pre-Experiment Screening

Prior to the experiments, all participants took our stereoscopic acuity and color vision tests to make sure they had normal stereoscopic and color vision. In the stereoscopic acuity test, participants viewed two side-by-side RDS sinusoidal corrugation stimuli. Each trial commenced with a 1000 ms noise phase to eliminate the influence of the previous depth stimuli, followed by the presentation of static Test Stimuli. The Standard Stimulus (randomly positioned on the left or right) had a fixed peak amplitude of 6.3 arcmin, while the Test Stimulus had one of 9 varying peak amplitude ratios relative to the standard (0.77, 0.82, 0.88, 0.94, 1.00, 1.06, 1.12, 1.18, and 1.23). Participants were required to indicate which stimulus appeared to have larger depth via a key press. There was no feedback about whether their choice was correct or not during the test. Each participant completed 4 repetitions for each of the 9 depth levels, totaling 36 trials. A minimum accuracy rate of 65% was required to qualify for the study. Additionally, color vision was assessed using the color vision test plates. Those who could not read the number or read the wrong number were excluded from the experiments. All recruited participants successfully passed both the stereoscopic and color vision tests.

### 3.2. Experiment 1: Influence of Different Motion Speeds of the Observed Target on Depth Perception

**Figure 2:**
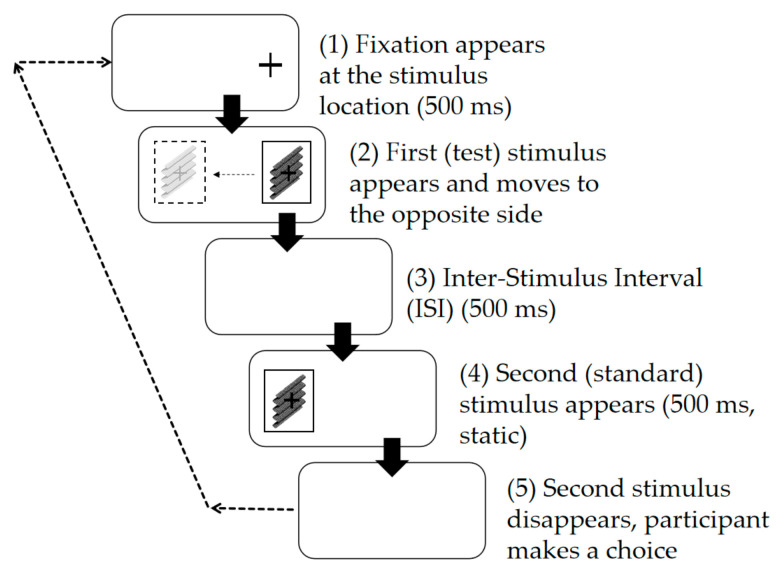
Schematic diagram of experimental procedure for motion scenario in Experiment 1.

**Figure 3:**
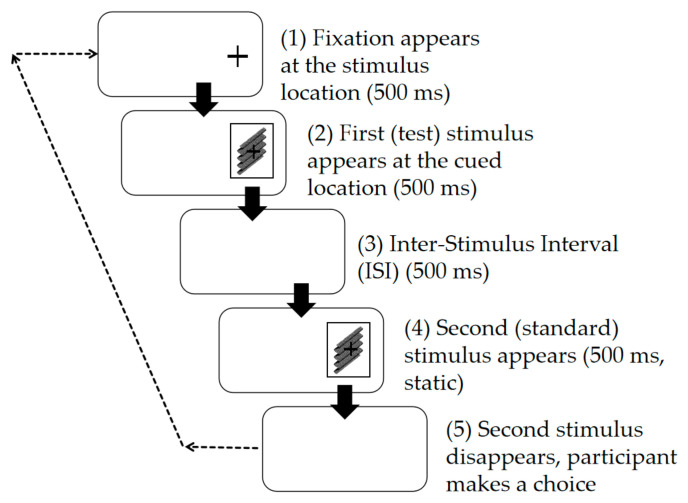
Schematic diagram of the experimental procedure for the static scenario in Experiment 1.

Experiment 1 primarily investigated the effect of different motion speeds of the observed target on depth perception. There were four conditions in Experiment 1: 0 (static), 5.0 arcmin/s, 10.0 arcmin/s, and 15.0 arcmin/s. In each condition, the stimulus exhibited only one motion speed. The conditions where the motion speed was not 0 are referred to as “motion scenarios” (Figure 2), and the condition where the speed was 0 is referred to as the “static scenario” (Figure 3) [22].

The procedure for the motion scenario is shown in Figure 2. Before the experiment began, the participant sat 57 cm from the screen. Once ready, the participant pressed a designated start key (e.g., Spacebar) to trigger the stimulus. A fixation cross appeared at the center of the left or right side of the screen, indicating the starting position of the upcoming stimulus. Subsequently, the first stimulus (the “Test Stimulus”) appeared at the fixation point and moved toward the other side of the screen at one of the aforementioned speeds. After the center of the image moved to the center of the other side of the screen, the image disappeared. Then, after an inter-stimulus interval (ISI) of 500 ms, a second stimulus (the “Standard Stimulus”) appeared at the position where the Test Stimulus disappeared and was presented for 500 ms before disappearing. Finally, the participant judged which stimulus (Test or Standard) had a larger depth and made a selection via the keyboard.

Unlike the motion scenario, in the static scenario (Figure 3), the Test Stimulus was also presented statically and displayed for only 500 ms. After an ISI of 500 ms interval, the Standard Stimulus appeared at the position where the Test Stimulus disappeared and was presented for 500 ms. In the experiment, the side of the screen from which the stimulus appeared was randomized, preventing prediction by the participant.

The experiment employed the Method of Constant Stimuli using a two-alternative forced-choice (2AFC) paradigm. The peak amplitude of the Standard Stimulus was a fixed value of 6.3 arcmin. The peak depth values of the Test Stimulus were 0.77, 0.82, 0.88, 0.94, 1.00, 1.06, 1.12, 1.18, and 1.23 times the Standard Stimulus (these ratios are referred to as “depth ratios”). The 9 different depth values appeared randomly, and participants could not predict the depth of the next image. Each participant performed 8 repetitions for each depth value, resulting in a total of 9 (depth ratios) × 8 (repetitions) × 4 (conditions) = 288 trials. The experiment was divided into two phases conducted on different days.

### 3.3. Experiment 2: Influence of Different Colors of the Observed Target on Depth Perception

Experiment 2 focused on the influence of different colors of the observed target on depth perception. In contrast to Experiment 1, this experiment selected four colors of corrugation stimuli: Red, Green, Blue, and White. The calibrated CIE 1931 xy chromaticity coordinates were (0.409, 0.325), (0.284, 0.405), (0.229, 0.254), and (0.313, 0.330), respectively. Each color stimulus had two motion speeds: 10.0 arcmin/s and 20.0 arcmin/s. The white corrugation stimulus under the static scenario served as the control group. The depth amplitude of the stimuli was the same as in Experiment 1 [23].

The experimental procedure was identical to that of Experiment 1 (Figure 2). In each trial, the color of the Test Stimulus was the same as that of the Standard Stimulus. The presentation timing was the same as in Experiment 1. The Experiment 2 conditions were defined as 4 (Colors) × 2 (Speeds) = 8. Each participant completed a total of 9 (depth ratios) × 8 (repetitions) × 8 (conditions) = 576 trials.

### 3.4. Experiment 3: Influence of Different Positions of the Observed Target on Depth Perception

**Figure 4:**
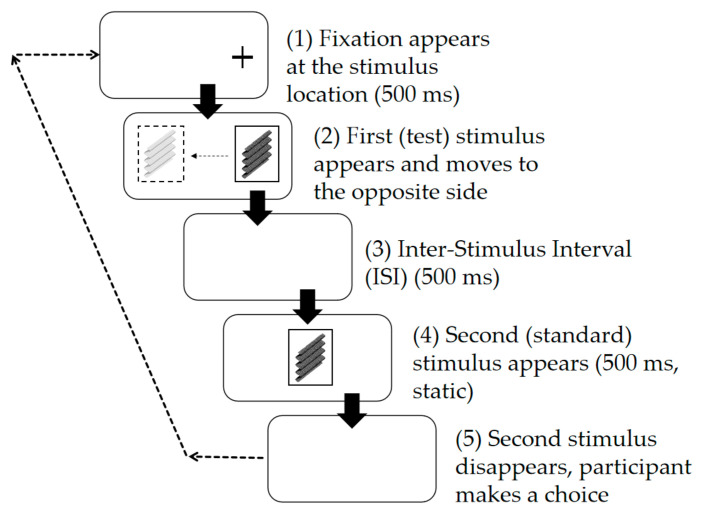
Schematic diagram of the experimental procedure for Experiment 3.

To investigate the effect of spatial position on depth perception, Experiment 3 introduced a position variable with two conditions: “random position” and “central position.” In the random-position condition, the Test Stimulus and Standard Stimulus were the same as those designed in Experiment 1, which were designed as a control group, while in the central-position condition, as shown in Figure 4, the key modifications were the fixation protocol and stimulus positioning. As in Experiment 1, at the beginning of each trial, a Nonius fixation appeared at the center of the left or right side of the screen for 500 ms to indicate where the Test Stimulus would appear. Subsequently, the Test Stimulus appeared at the cued location. Unlike Experiment 1, there was no fixation cross at the center of the Test Stimulus; instead, participants were asked to keep their gaze on the stimuli and track the movement. Following the disappearance of the Test Stimulus, there was a 500 ms blank interval. Then, the Standard Stimulus always appeared at the center of the screen (which was also different from Experiment 1) for 500 ms (static), after which participants would make their choice. The experiment included three motion speeds (0 (static), 5.0 arcmin/s, and 10.0 arcmin/s) and two spatial positions (random position and central position), totaling six experimental conditions (3 speeds × 2 positions). Each participant completed a total of 9 (depth ratios) × 8 (repetitions) × 6 (conditions) = 432 trials. The stimulus color was white.

## 4. Results

### 4.1. Experiment 1: Influence of Different Motion Speeds on Depth Perception

**Figure 5:**
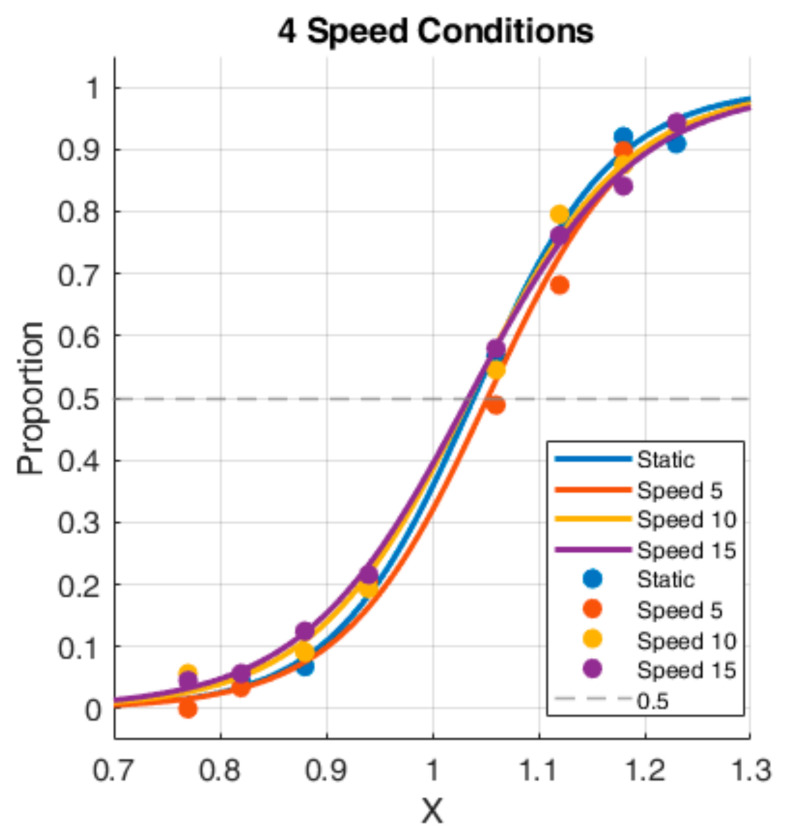
Sigmoid functions for the four scenarios in Experiment 1.

Based on the key-press responses in Experiment 1, the proportion of trials where all participants perceived the Test Stimulus as having a larger peak value was calculated. Sigmoid curves were fitted to this data, as shown in Figure 5. The horizontal axis represents the depth ratio, and the vertical axis represents the proportion of the Test Stimulus being perceived as “having larger depth” (ranging from 0 to 1). The blue, red, yellow, and purple curves represent the fitted curves for scenarios with speeds of 0 (static), 5.0 arcmin/s, 10.0 arcmin/s, and 15.0 arcmin/s, respectively.

**Figure 6:**
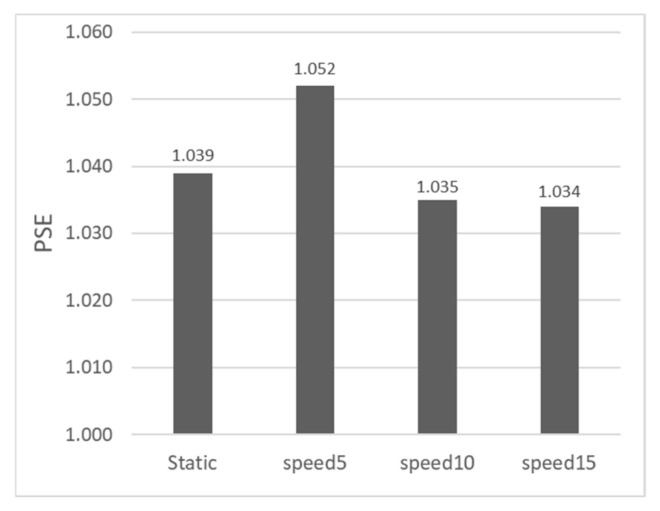
Distribution of PSEs for the 4 experimental conditions in Experiment 1.

For each participant, the Point of Subjective Equality (PSE) was calculated (the depth ratio value on the horizontal axis corresponding to 0.5 on the vertical axis). The distribution of PSEs for participants under each condition was obtained through repeated-measures analysis of variance (ANOVA) (Figure 6).

As seen in Figure 6, the PSE for the static scenario is 1.039, indicating that participants perceived the peaks as equal when the Test Stimulus peak was 1.039 times that of the Standard Stimulus. Compared to the static scenario, the PSE for the 5.0 arcmin/s speed is 1.052, suggesting that at lower speeds, motion parallax might have a potential inhibitory effect on depth perception. Furthermore, the time required for the stimulus to move from one side of the screen to the other is relatively long, during which factors such as motion aftereffect might have an influence. When the motion speed was higher (10.0 arcmin/s and 15.0 arcmin/s), motion parallax had a potential promoting effect on depth perception, resulting in PSE values slightly lower than those in the static scenario.

In Experiment 1, we used ANOVA to examine the effect of the four motion speed conditions on depth perception. The ANOVA results showed that the main effect of speed was not significant $\left(F(3,30)=0.468,\;p\;=0.707,\;\eta_{p}^{2}=0.045\right)$. Moreover, post hoc pairwise comparisons with Bonferroni correction were performed. The results revealed no statistically significant differences between any speed conditions (all adjusted p ≥0.641, 95% confidence intervals (CIs) included zero). This result indicates that within the speed range set in this experiment, changing the target’s motion speed did not significantly affect the participants’ depth perception thresholds overall.

**Figure 7:**
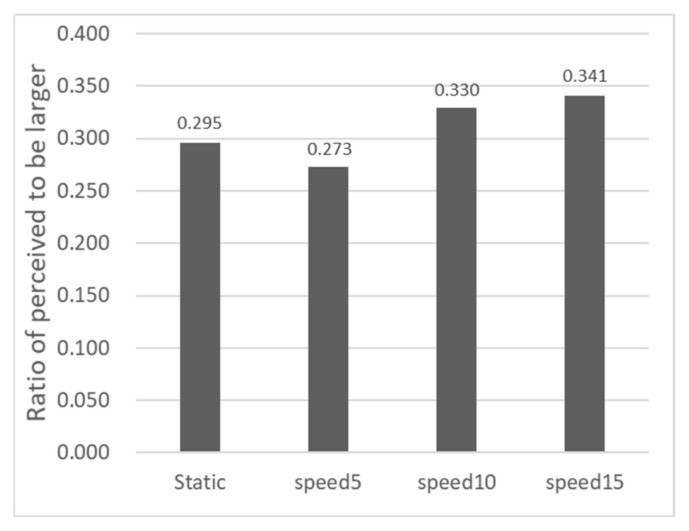
Proportion of trials where the Test Stimulus was perceived as having a larger depth when the depth ratio equaled 1 in Experiment 1.

Figure 7 displays the proportion of participants perceiving the Test Stimulus as deeper when the depth ratio was equal to 1 in Experiment 1. Compared to the static scenario, the proportion decreased at 5.0 arcmin/s, suggesting a potential inhibitory trend of motion parallax at this speed. Conversely, at the other two speeds (10.0 arcmin/s and 15.0 arcmin/s), the proportion increased, suggesting a potential promoting trend. This is consistent with the PSE distribution results in Figure 6. However, an ANOVA conducted on the data where the depth ratio equaled 1 showed no significant main effect of speed $\left(F(3,30)=0.45,\;p\;=0.721,\;\eta_{p}^{2}=0.043\right)$. Post hoc tests further confirmed this lack of statistical significance, showing no detectable differences between the static baseline and any motion condition (all adjusted p =1.000, 95% CIs included zero). This confirms that when the Test and Standard Stimuli are physically equal in peak value, simply changing the target’s motion speed does not significantly alter the proportion of trials where the Test Stimulus is perceived as deeper.

### 4.2. Experiment 2: Influence of Different Colors on Depth Perception

**Figure 8:**
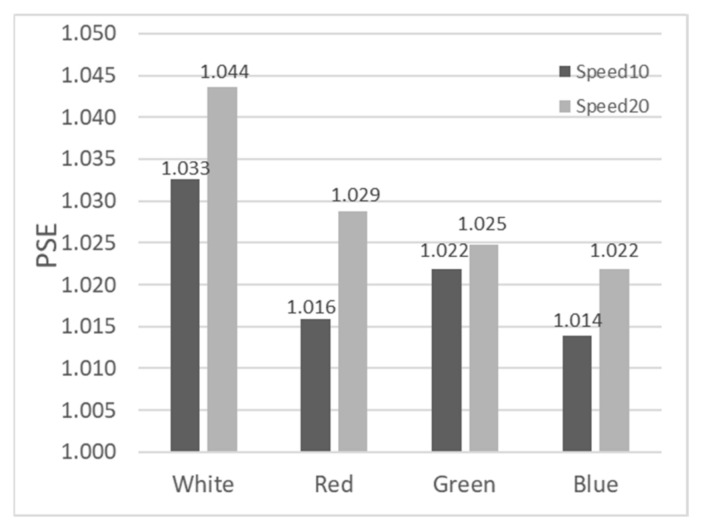
Distribution of PSEs for the 8 experimental conditions in Experiment 2.

Based on participant feedback in Experiment 2, Sigmoid functions were fitted for the four color targets (White, Red, Green, Blue) under two motion speeds (10.0 arcmin/s and 20.0 arcmin/s). PSE values were extracted using the same method as in Experiment 1, with the results shown in Figure 8.

Comparing the PSE values of the white stimulus in the static scenario from Figure 5 (1.039), the PSE values for red, green, and blue stimuli in the 10.0 arcmin/s and 20.0 arcmin/s motion scenarios all decreased to varying degrees, indicating that motion parallax has a promoting effect on depth perception. In the motion scenarios, the PSE values for red, green, and blue stimuli were slightly lower than or equal to that of the white stimulus, suggesting that color also aids in enhancing depth perception to a certain extent. When the speed increased from 10.0 arcmin/s to 20.0 arcmin/s, the PSE values for all four color conditions increased, indicating that increasing motion parallax at this stage had a certain inhibitory effect on depth perception.

In Experiment 2, we used a 2 (Speed: 10.0 arcmin/s, 20.0 arcmin/s) × 4 (Color: White, Red, Green, Blue) ANOVA to examine the effects on depth perception (PSE). The results showed no significant main effect of speed $\left(F(1,9)=0.518,\;p\;=0.49,\;\eta_{p}^{2}=0.054\right)$. The main effect of color also did not reach significance $\left(F(3,27)=2.170,\;p\;=0.115,\;\eta_{p}^{2}=0.194\right)$. Moreover, we conducted post hoc pairwise comparisons for the color conditions. No significant differences were found between the white stimulus and the colored stimuli (e.g., white vs. red: p =0.569; white vs. blue: p =0.359; all 95% CIs included zero). Additionally, the speed × color interaction was not significant $\left(F(3,27)=0.262,\;p\;=0.852,\;\eta_{p}^{2}=0.028\right)$. This suggests that while color may have some influence on depth perception, this influence does not change significantly with variations in motion speed within the parameters of this experiment.

**Figure 9:**
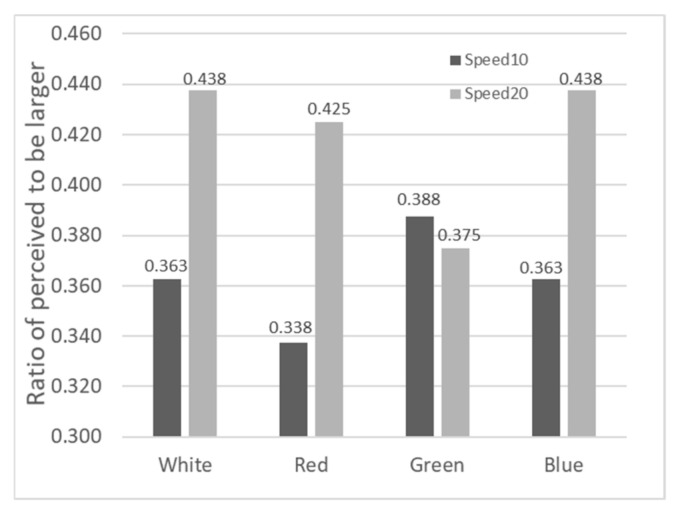
Proportion of trials where the Test Stimulus was perceived as having a larger depth when the depth ratio equaled 1 in Experiment 2.

Figure 9 shows the proportion of trials where the Test Stimulus was perceived as deeper when the depth ratio equaled 1 in Experiment 2. Dark bars represent 10.0 arcmin/s, and light bars represent 20.0 arcmin/s. The values range between 0.34 and 0.44. Compared to the static white stimulus ratio of 0.30 in Figure 7, the ratios in Figure 9 increased across all conditions, suggesting a potential promoting trend of motion speed and color on depth perception, consistent with the PSE results. The 2 (Speed) × 4 (Color) ANOVA results showed no significant main effect for speed $\left(F(1,9)=1.39,\;p\;=0.268,\;\eta_{p}^{2}=0.134\right)$, no significant main effect for color $\left(F(3,27)=0.08,\;p\;=0.972,\;\eta_{p}^{2}=0.008\right)$, and no significant interaction $\left(F(3,27)=0.41,\;p\;=0.745,\;\eta_{p}^{2}=0.044\right)$. Consistent with the omnibus ANOVA, post hoc analyses revealed no significant pairwise differences between any color conditions (all adjusted p =1.000, 95% CIs included zero).

### 4.3. Experiment 3: Influence of Different Positions on Depth Perception

**Figure 10:**
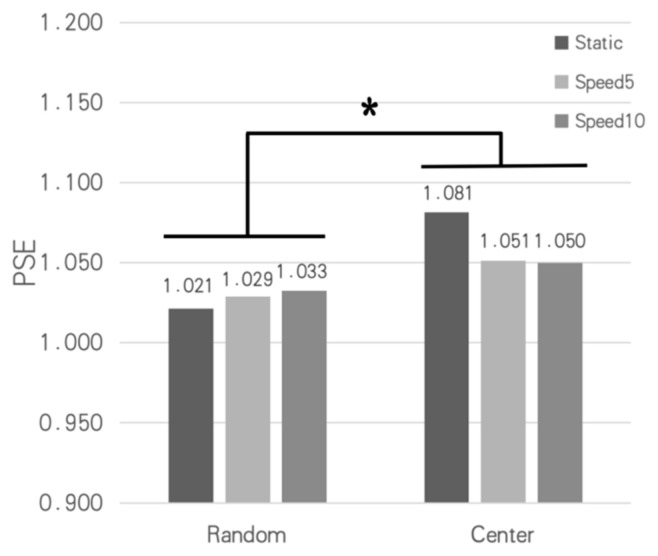
Distribution of PSEs for the 6 experimental conditions in Experiment 3. The asterisk (*) indicates a significant main effect of position based on a repeated-measures ANOVA (p <0.05).

Based on feedback from Experiment 3, Sigmoid fitting and PSE extraction were performed separately for the “random position” and “center position” conditions under three speeds. The results are shown in Figure 10. The three bars on the left represent PSE distributions for random positions at speeds of 0 (static), 5.0 arcmin/s, and 10.0 arcmin/s, while the three bars on the right represent center positions at the same speeds. It can be seen that the PSE values for the center-position condition are consistently higher than those for the random-position condition, indicating that participants were more inclined to perceive the Standard Stimulus as having a larger depth when the Standard Stimulus always appeared at the center of the screen (i.e., a different position from the Test Stimulus).

A 2 (position: random, center) × 3 (speed: 0 (static), 5.0 arcmin/s, 10.0 arcmin/s) ANOVA was conducted on the PSE values in Figure 10. The results indicated a significant main effect of position $\left(F(1,7)=14.61,\;p=0.007,\;\eta_{p}^{2}=0.676\right)$. Post hoc analysis for the main effect of position confirmed a significant difference between the random and center conditions (mean difference = −0.041, p=0.007, 95% CI [−0.066, −0.016]). The main effect of speed was not significant $\left(F(1.12,7.86)=0.01,\;p=0.987,\eta_{p}^{2}=0.002\right)$. However, the position × speed interaction was significant $\left(F(2,14)=4.74,\;p=0.027,\eta_{p}^{2}=0.404\right)$. This suggests that while changing speed alone did not significantly affect depth perception, there was a significant difference in depth perception when the Test and Standard Stimuli appeared at different screen positions compared to when they appeared at the same position. This implies that performing depth perception judgments when objects are at different positions may involve areas of higher-level brain function (such as attention and memory).

**Figure 11:**
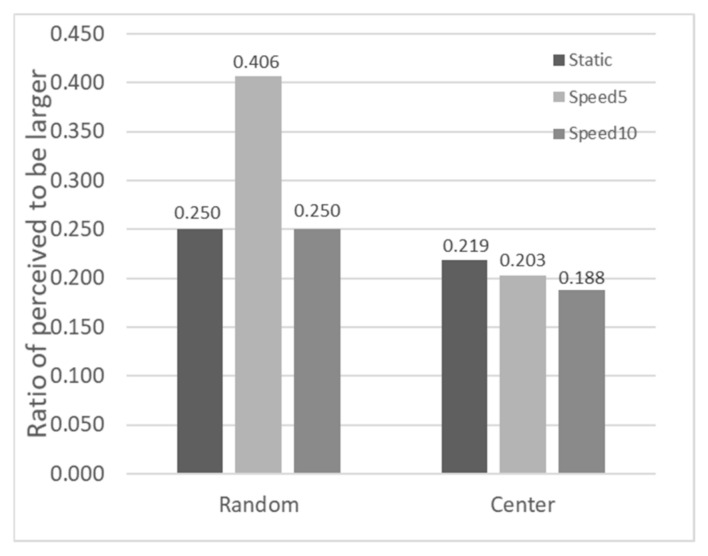
Proportion of trials where the Test Stimulus was perceived as having a larger depth when the depth ratio equaled 1 in Experiment 3.

Figure 11 analyzes the proportion of Test Stimuli perceived as deeper when the depth ratio equaled 1. The left three bars represent random positions, and the right three bars represent the center-position condition. A 2 (Position) × 3 (Speed) ANOVA showed no significant main effect of position $\left(F(1,7)=2.509,\;p=0.157,\;\eta_{p}^{2}=0.264\right)$, no significant main effect of speed $\left(F(2,14)=0.891,\;p=0.432,\;\eta_{p}^{2}=0.113\right)$, and no significant interaction $\left(F(2,14)=0.947,\;p=0.411,\;\eta_{p}^{2}=0.119\right)$. Post hoc comparisons further supported these findings, yielding no significant differences for position (p =0.157) or speed pairs (all adjusted p ≥0.442, 95% CIs including zero). This indicates that when the physical depth values generated by binocular disparity are equal, changing the stimulus position and speed within a certain range does not significantly affect depth perception judgments.

## 5. Discussion

### 5.1. Influence of Different Motion Speeds on Depth Perception

The results of Experiment 1 showed a trend where relatively high motion speeds (10.0 and 15.0 arcmin/s) tended to promote greater perceived depth compared to the static condition, whereas low speed (5.0 arcmin/s) tended to inhibit it. This pattern suggests that the interaction of motion parallax and binocular disparity cues on depth perception is velocity-dependent. This finding is consistent with Holmin and Nawrot’s results. In their study, they proposed that the perceived depth was optimal only within a specific velocity window and deteriorated outside this range due to unreliable signals [24]. Furthermore, the inhibitory trend observed at low speed (5.0 arcmin/s) aligned with the observation by Ujike and Ono. They suggested that at lower velocities, the motion signal was too weak to be effectively utilized by the visual system, and thus failed to enhance depth perception [25].

### 5.2. Influence of Different Colors on Depth Perception

In Experiment 2, although the main effect of color was not statistically significant, there was a consistent trend where red targets resulted in lower PSE values (promoting perceived depth) compared to white or blue targets. This observation agrees with the classical hierarchy of chromostereopsis described by Egusa, who found that long-wavelength color (e.g., Red) was most frequently perceived as being nearer [26].

However, the result in our study whereby color did not produce a statistically significant independent effect could be explained by the study of Guibal and Dresp, in which they argued that color was not an independent depth cue, but influenced by luminance contrast and stimulus geometry. Moreover, Thompson also stated that the perceived depth seen in chromostereopsis might often be the combination of a luminance-based depth effect and a color-based depth effect [27].

### 5.3. Influence of Different Positions on Depth Perception

The results of Experiment 3 revealed a significant difference in perceived depth between the random- and central-position conditions. Specifically, participants perceived the Standard Stimulus as having significantly larger depth (higher PSE) when it appeared at the central position on the screen (a different location to that in the preceding Test Stimulus) compared to when it appeared at the random position (the same location as in the preceding Test Stimulus).

In the random-position condition, the Standard Stimulus appeared at the same spatial location as the preceding Test Stimulus, separated by an ISI of 500 ms. One explanation for this spatiotemporal configuration was the mechanism of Inhibition of Return (IOR). IOR refers to a phenomenon where, after attention is drawn to a stimulus, the response to a subsequent target appearing at the same location is delayed or inhibited once a specific interval has passed [28]. Due to this inhibitory effect, the perceived depth of the Standard Stimulus was underestimated. Consequently, the Test Stimulus required less physical depth to subjectively match the inhibited Standard Stimulus, resulting in a lower PSE. In contrast, in the central-position condition, the Standard Stimulus appeared in a previously unoccupied location. According to Yantis and Jonides, a sudden onset at a new location captures attention and is processed with priority, thereby enhancing the clarity and intensity of its perception [29]. Consequently, the Test Stimulus in this condition required larger physical depth to achieve subjective equality, leading to a higher PSE.

### 5.4. Innovations and Limitations

This study presented three groups of psychophysical experiments to clarify the mechanisms of human depth perception and the influences of different motion speeds, colors, and positions of the stimuli. The first novel point was the design of a stereoscopic random-dot sinusoidal stimulus paradigm, which effectively incorporated motion parallax, color, and spatial position variables into the dominant framework of binocular disparity. This allowed for a systematic investigation of how these monocular cues interact with perceived depth in a controlled environment. The second key innovation was the significant difference in perceived depth induced by the same and different positions between the Test and Standard Stimuli. By revealing that depth perception is significantly enhanced when the Standard Stimulus appears at a new location compared to a repeated location, this study suggests the possibility of high-level cognitive involvement (like visual attention) in depth perception. These findings offer a theoretical basis for enhancing depth realism in VR and AR displays by optimizing stimulus placement and motion parameters.

However, there are still some limitations to this study. First, the sample size is relatively small, and the ranges of colors and motion speeds of the stimuli are small. Second, in the position experiment, the absence of precise eye-tracking data presents a constraint. Future research can expand the color and velocity ranges and combine eye tracking and electrophysiology techniques to obtain multi-dimensional experimental data and construct models with higher accuracy.

## 6. Conclusions

Against the background of 3D display applications such as virtual reality, this study systematically investigated the influence and interaction of three cues—motion parallax, color, and stimulus position—on depth perception using random-dot stereograms constructed based on binocular disparity cues. The experimental results show that when motion parallax is introduced to random-dot stereogram stimuli, a small amount of motion parallax (speed of 5.0 arcmin/s) exerts a certain inhibitory effect on depth perception, whereas larger motion parallax (speeds of 10.0 arcmin/s and 15.0 arcmin/s) produces a certain promoting effect. Introducing red, green, or blue colors to the moving stimuli also yields a certain degree of promotion in depth perception. Furthermore, when the positions of the sequentially presented Test Stimulus and Standard Stimulus differ, it significantly impacts depth perception; this process likely involves areas of higher-level brain function (such as attention). Thus, when multiple visual cues appear simultaneously, their influence on depth perception might involve complex interactions, and even areas of higher-level brain function. These findings provide data support for the rendering of depth images in VR, AR, and 3D displays, promoting the enhancement of depth realism in such products.

## Data Availability

The data presented in this study are available on request from the corresponding author.
